# Supplementary figures and images for: Genetic characterization of a mild isolate of papaya ringspot virus type-P (PRSV-P) and assessment of its cross-protection potential under greenhouse and field conditions
Source: PLoS One. 2021 Feb 5;16(2):e0241652. doi: 10.1371/journal.pone.0241652 (PMC7864462; doi:10.1371/journal.pone.0241652)

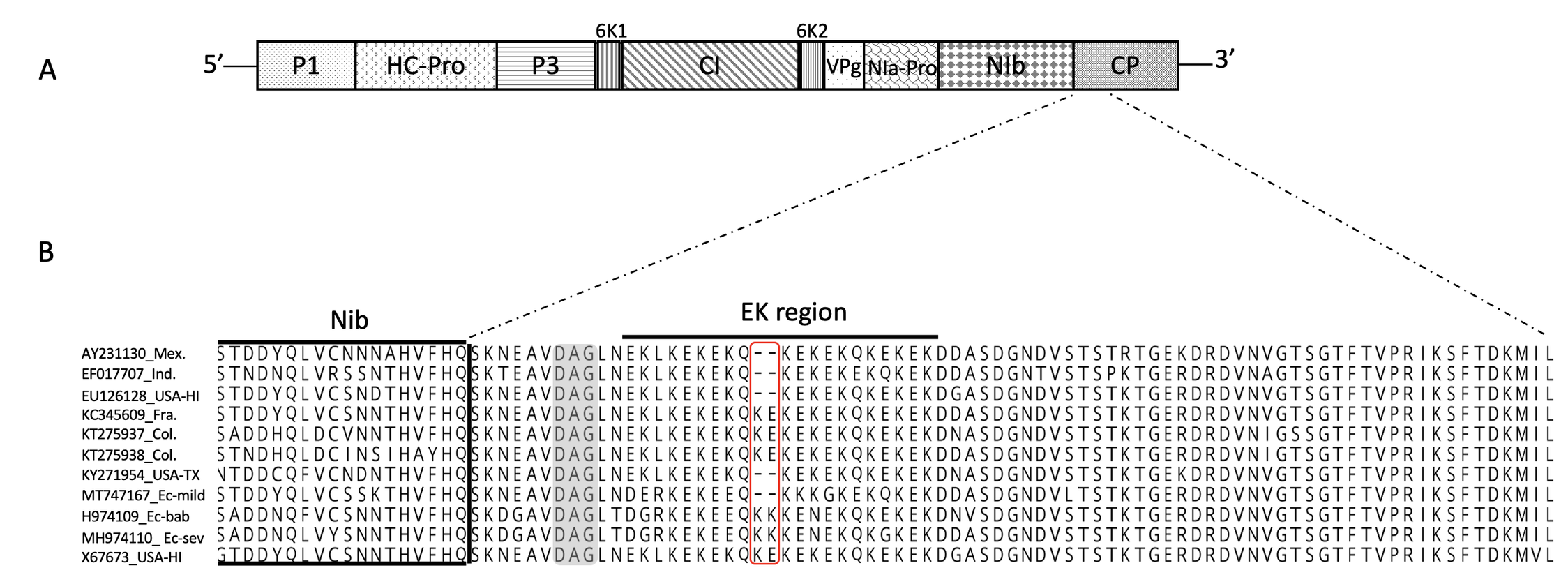

Supplement: S1 Fig — A) Canonical genome organization of PRSV. B) A fraction of the coat protein (CP) alignment across related taxa, including sequences from Ecuador (Ec), Mexico (Mex), Hawaii (USA-HI), Texas (USA-Tx), Colombia (Col) and India (Ind). The aphid transmission determinant ‘DAG’ motif and the EK region are indicated, where the mild isolate from Ecuador, along with four additional isolates from the USA, Mexico and India, lack two amino acids. NCBI accession numbers are shown on the left. (TIF) [file pone.0241652.s001.tif]

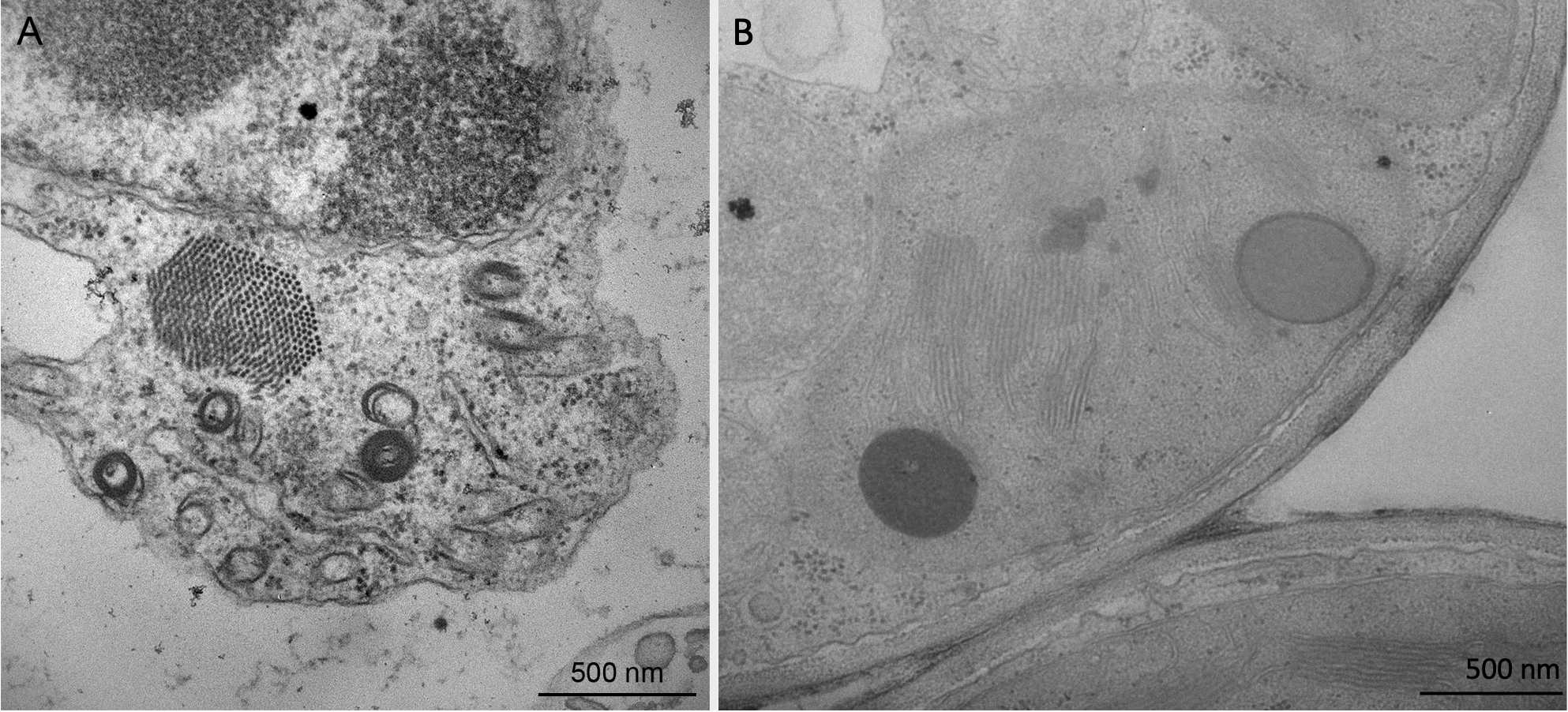

Supplement: S2 Fig — A) The cytoplasm of leaf mesophyll cells infected with the severe isolate of papaya ringspot virus (PRSV-sev) showing pinwheel inclusions next to filamentous virus particles. B) The cytoplasm of leaf mesophyll cells infected with the mild isolate of papaya ringspot virus (PRSV-mild) where no pinwheels inclusions or filamentous particles are observed. (TIF) [file pone.0241652.s002.tif]

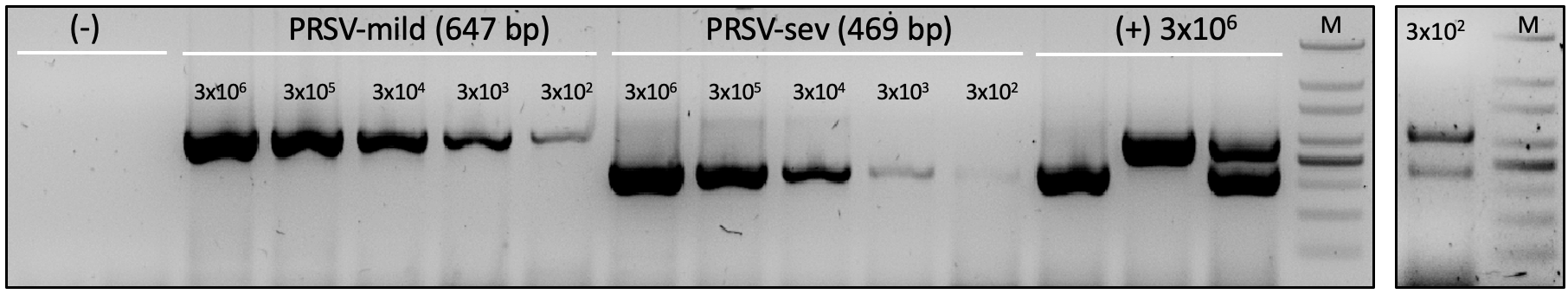

Supplement: S3 Fig — The size in base-pairs (bp) of each amplification product is shown. Sensitivity and specificity for each primer set was tested in single- and duplex. In vitro RNA dilutions started from an initial amount of 3 x 10^6^ copies for each target (1ul) resuspended in 9 ul of virus-free papaya total RNA at a concentration of 100 ng/ul. Ten-fold dilutions followed using this target-total RNA mixture in molecular biology grade water and used as template (1ul) for reverse transcription in a 10 ul reaction. M: DNA 100bp ladder. (+) positive control (-) negative controls. (TIF) [file pone.0241652.s003.tif]

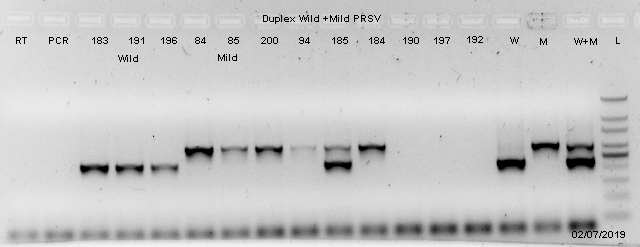

Supplement: S1 File — (JPG) [file pone.0241652.s004.jpg]

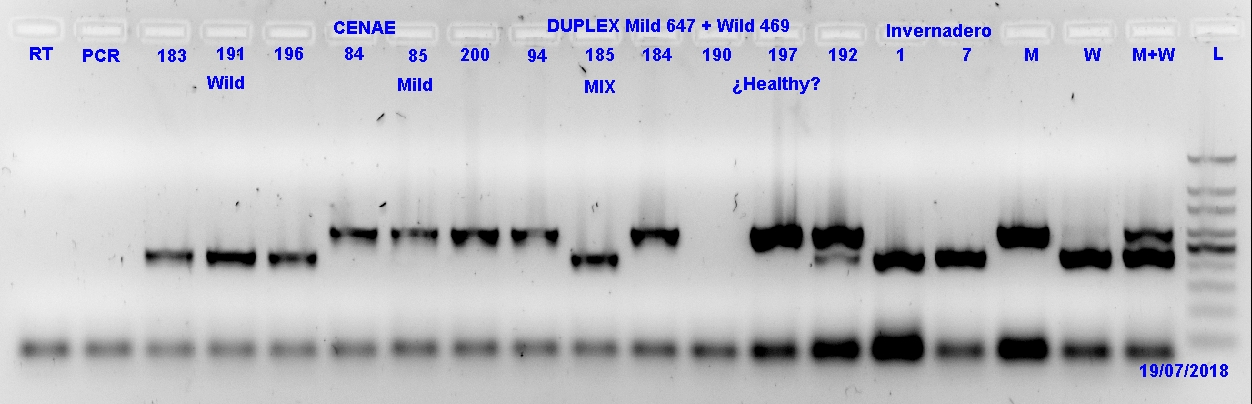

Supplement: S2 File — (JPG) [file pone.0241652.s005.jpg]

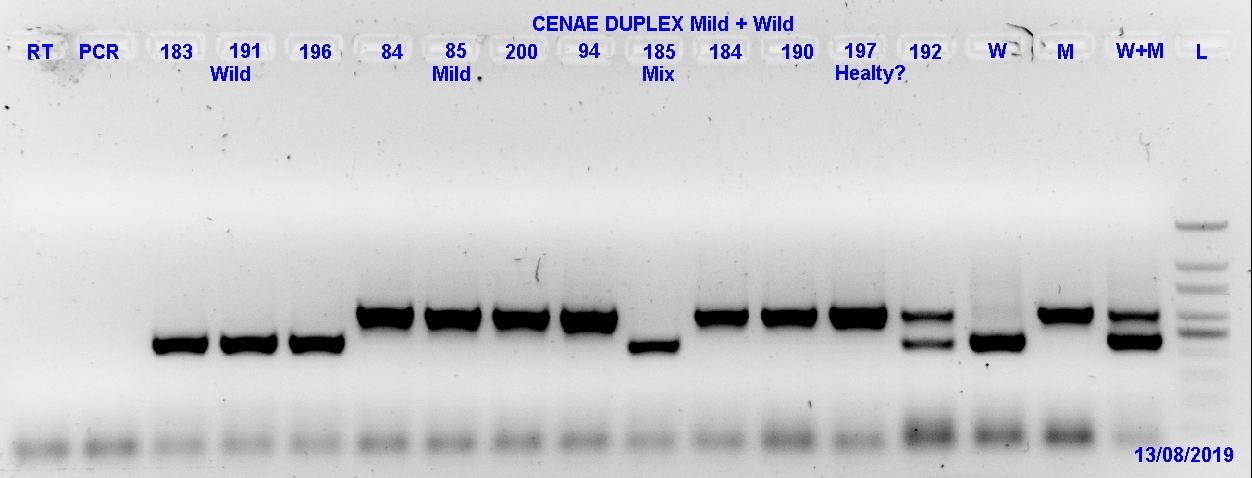

Supplement: S3 File — (JPG) [file pone.0241652.s006.jpg]

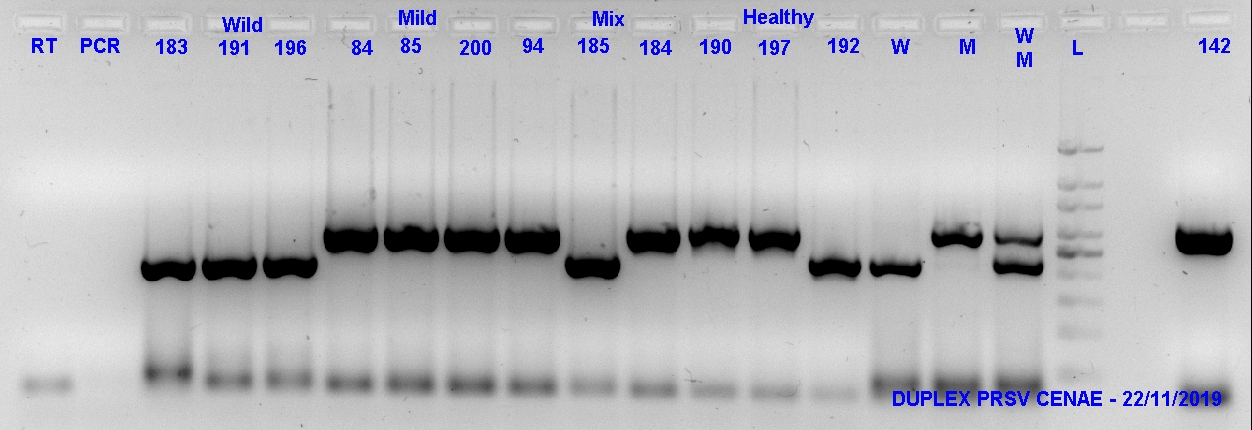

Supplement: S4 File — (JPG) [file pone.0241652.s007.jpg]

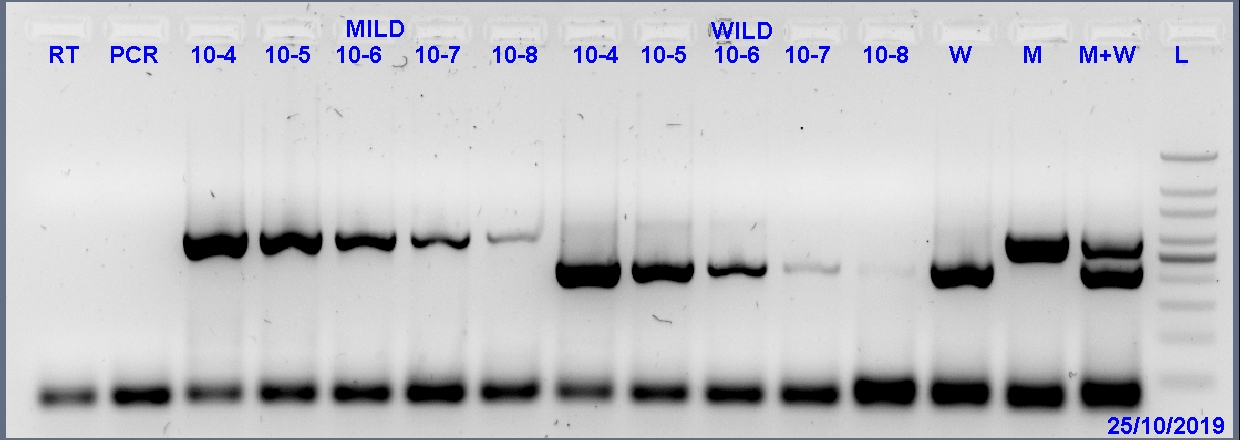

Supplement: S5 File — (JPG) [file pone.0241652.s008.jpg]

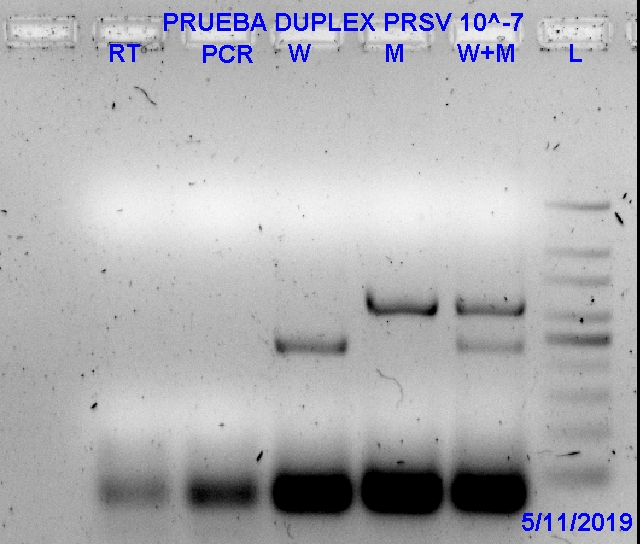

Supplement: S6 File — (JPG) [file pone.0241652.s009.jpg]
